# A Feature of Recent Progress

**Published:** 1921-07-30

**Authors:** 


### PROTEIN INJECTION IN TREATMENT.

### A Feature of Recent Progress.

The past year has not been medically remarkable for any really outstanding therapeutic discoveries, and after the rush of the war period it is, perhaps, not surprising that most of our time has been spent upon consolidating the progress then made. There is one exception, however, in the case of the so-called protein therapy.

There is no doubt that a number of unexpected clinical results, in some cases cures, have been produced by the subcutaneous injection of protein substances having no apparent or specific connection with the disease under consideration. The sole connection has been that a foreign protein substance is introduced, and the human body, a protein complex, has responded in a way that produces reparative activity not only in the healthy, but, more important, in diseased tissues also.

The most general explanation is that no new function of any organ is produced. The introduction of a non-specific protein simply produces a universal stimulating action on all cellular elements. This may be exhibited either actively or passively. The latter results simply in the rapid removal of detrimental cleavage products in exhaustion states, and never, therefore, results in more than recovery of normal activity in a weakened organ. "Active" activation occurs, on the other hand, in non-exhaustion states, and may result in the hyper-activity of an organ and in indirect benefit from the cause.

Be that as it may, it is an unquestioned fact that injection of protein does have a decidedly stimulating effect. Its magnitude, however, differs widely with the particular protein used. It is found, for example, that globulous fibrins, albumens, and nucleo-albumens produce but little effect in single injections. In contrast, histones and protamines produce intense reactions.

And this brings us to an interesting point. The latter normally occur in the body only in combination with nucleic acid, which conjugation forms are non-toxic. Also the simple known amino-acids and low molecular peptones of metabolism are non-toxic; but as we ascend the chemical scale to the more complicated peptones the toxicity increases, until we reach the observed effects of bacterial proteins.

Therapeutically speaking, all this indicates that the selection of our protein might possibly, by its chemical composition, be nicely graded to the effect we require. No doubt this stage will ultimately arrive, but meanwhile it is a remarkable illustration of the close parallel between structural chemical composition of substances and their pathogenic power towards man.

### Individual Sensitiveness to Protein.

Meanwhile, closely connected with the possibility of deliberate stimulation by foreign protein we have the many observations made upon personal idiosyncrasies to the effects of a variety of simple proteins met with in everyday life. The hyper-sensitiveness is not confined to animal proteins—*e.g.*, horse dander—for the vegetable world is equally productive in this respect, and we have the classical example of so-called hay fever. It is nowadays a relatively simple experiment to inject into a person's arm small quantities of the various possible proteins and observe which, if any, produce an untoward reaction. In practice it is remarkable how many unsuspected cases and causes have in this way been brought to light. To mention but the asthmatics, the varieties of dermatitis, and recurrent alimentary "catarrhs" is to tell but a part of the story. In many hospitals a routine examination of suspected cases is already being carried out on these lines. If one can detect which is the irritant protein, then it is relatively easy, by a series of steadily increasing injections, to de-sensitise the patient against its effects. Thus we have here a perfectly sound therapeutic measure, but are, at the same time, completely in the dark as to why the particular protein should ever have irritated the particular person. Perhaps it is, in both, again a question of detailed chemical structure. But at present we can only surmise.

### Peptone Injections.

In any case, as it is definitely established that a general protein stimulation can be produced by injecting into the body another substance of this class, it is interesting to note some of the results already produced. We can omit the use of non-specific vaccines—*e.g.*, the T.A.B. injection of the war days, as afterwards used in huge doses for entirely other infections. Here the principle was simply protein injection, a non-specific bacterial protein being chosen. More typical is the use of peptone. The introduction of peptone in any quantity produces all the phenomena of shock. Many authorities hold that peptone shock and what we have for long called anaphylactic shock are identical. If this shock is produced in small quantities at a time, one may, broadly speaking, view the effect simply as a beneficial protein stimulation throughout the patient's body. In practice intravenous injection is given very slowly, and during the whole period the pulse is carefully watched. If the pulse-rate increases injection is immediately stopped, and so on. Usually, with these precautions, no immediate symptoms occur beyond transient palpitation and rapid breathing, but one or two hours later there is a marked reaction, accompanied by sweating and even rigors. This again is quite transitory, and the experts claim that the whole protein and metabolic systems of the body are in this way brought to increased activity. It is impossible to go into their theories of the exact mechanism, but, as might in these times be expected, the greatest success has been obtained with bacterial diseases, particularly with septicæmia, where the organisms are resisting the endeavours of body proteins to destroy them.

A variety of hydrolysed vegetable proteins have been somewhat similarly used under the name of "proteals." In this case the stimulation appears to be directed against two main systems, the nutritional and the blood. In any case, it seems to be definitely established that many irregular blood conditions become under its influence more normal. In anæmia, red corpuscles increase in number and in individual hæmoglobin content. An increased white count is often reduced, and the current theories account for the effect by claiming a direct effect on the blood-forming system. A stimulus is certainly produced, but whether direct or indirect it is as yet impossible to say. On the same somewhat uncertain lines a number of attempts have been made to employ subcutaneous injections of milk. A great variety of benefits have been claimed, but it is a remarkable fact that fresh milk which is approximately sterile or fresh breast milk produces no reaction whatever. The suggestion is, therefore, very natural that the reactions which *have* been produced arose from the bacterial toxins and decomposition products commonly present in most milk samples. Whichever way it is, one gives the instance as illustrative of the peculiar difficulties which surround a form of treatment still in its infancy. That protein shock can, in its artificial form, be turned to valuable therapeutic use there seems to be little doubt. But we must, though it is one of the most recent of our advances, still await the hour of its perfection.

